# Colorimetric Characterization of Color Imaging System Based on Kernel Partial Least Squares

**DOI:** 10.3390/s23125706

**Published:** 2023-06-19

**Authors:** Siyu Zhao, Lu Liu, Zibing Feng, Ningfang Liao, Qiang Liu, Xufen Xie

**Affiliations:** 1 School of Information Science and Engineering, Dalian Polytechnic University, Dalian 116034, China; zhaosiyudlpu@126.com (S.Z.); zibingfengdlpu@126.com (Z.F.); 2 National Key Lab of Colour Science and Engineering, Beijing Institute of Technology, Beijing 100081, China; liaonf@bit.edu.cn; 3 Research Center of Graphic Communication, Printing and Packaging, Wuhan University, Wuhan 430079, China

**Keywords:** color imaging system, colorimetric characterization, color space conversion, partial least squares

## Abstract

Colorimetric characterization is the basis of color information management in color imaging systems. In this paper, we propose a colorimetric characterization method based on kernel partial least squares (KPLS) for color imaging systems. This method takes the kernel function expansion of the three-channel response values (RGB) in the device-dependent space of the imaging system as input feature vectors, and CIE-1931 XYZ as output vectors. We first establish a KPLS color-characterization model for color imaging systems. Then we determine the hyperparameters based on nested cross validation and grid search; a color space transformation model is realized. The proposed model is validated with experiments. The CIELAB, CIELUV and CIEDE2000 color differences are used as evaluation metrics. The results of the nested cross validation test for the ColorChecker SG chart show that the proposed model is superior to the weighted nonlinear regression model and the neural network model. The method proposed in this paper has good prediction accuracy.

## 1. Introduction

Color imaging systems have been widely used in industrial manufacturing, medicine, geology exploration, art and other fields. Color characterization [1,2,3] is an important means to evaluate the color characteristics of color imaging systems; it establishes the transformation relationship between the device-dependent RGB color space and the device-independent color space of the color imaging systems, and realizes the color consistency from color information acquisition to color information output [4]. Colorimetric characterization is very important for image high-fidelity display, colorimetric measurement, color reproduction, color analysis, color management of different devices [5] and color appearance prediction [6], and the requirements for the accuracy and stability of color characterization are increasing for different applications. Color analysis [7,8,9,10] plays a very important role in the evaluation of the performance of many technologies including paper-based devices that use colorimetric reactions and that researchers have used with respect to different color spaces such as weighted RGB, greyscale, etc.

According to international standards, color characterization can be generally divided into two categories: spectral response-based and target color-based characterization methods [11,12,13]. The spectral response-based characterization method is to find out the relationship between the spectral response and the CIE color-matching function under the condition of knowing the three-channel spectral response of the color imaging system, and establish the transformation relationship between the device-dependent RGB color space and the device-independent CIEXYZ color space [14]. However, measurement for the three-channel spectral response of the imaging system not only requires special experimental equipment and measurement equipment, but also requires professional measurement methods [15]. Monochromators and radiometers are among the measurement equipment used, and experts are required to manage the operation in the laboratory [16]; the operation is difficult for general device users. The advantage is that if the spectral response is known, the tristimulus values of objects under any known spectral conditions of a light source can be predicted, and the estimation methods of camera spectral response characteristics are also constantly explored [17]. The target color-based color-characterization method establishes the mapping relationship between the input and output color spaces based on samples, including interpolation methods, regression models, etc. The method has low requirements for experiments and is widely used in practice. In the early stages of color characterization, interpolation methods were widely used [18]. One of the common color-characterization methods at that time was the three-dimensional lookup table method [19,20]. Based on different regression models, common target color-based color-characterization methods include least squares polynomial regression method [21,22], neural network [23,24,25,26,27], support vector machine [28], etc.; from the perspective of input features of the model, these methods can also incorporate different feature-selection and optimization techniques. RGB cross-polynomial features have been widely used in color measurement, information management and color correction for their high conversion accuracy and low computational cost [29,30,31,32,33]. Essentially, this is a technique that uses the nonlinearity of input features to perform dimensionality increase. In addition to polynomial expansion, other kernel-based increasing dimension techniques also need further exploration in the field of colorimetric characterization. There are many research methods based on the ordinary least squares method with RGB cross-polynomial dimensionality increase. In 2000, Hong et al. proposed a polynomial regression method [34], which uses different combinations of multiple inputs of R, G and B cross-terms. In 2007, Bianco proposed a pattern search optimization algorithm [35] to study the conversion from RGB to CIEXYZ space. It uses pattern search optimization of the least squares method for 3×3 RGB to XYZ conversion. Meanwhile, for the problems of medium color spectral reflectance reconstruction and training sample validity in color measurement, various improved algorithms of the ordinary least squares method have also been applied in different studies. In terms of color spectral reconstruction, in 2010, Shen et al. proposed that the partial least squares regression (PLS) method can also be used to build a regression model based on the correlation between response values and spectral reflectance [36]. In 2013, Heikkinen et al. proposed a kernel ridge regression (KRR) method for spectral reflectance [37]. Essentially, KRR is a method that nonlinearly transforms low-dimensional camera responses to high-dimensional feature space; and it performs regularized least squares regression on the reflectance data in the feature space. In 2019, Xiao et al. proposed a new method for spectral reflectance reconstruction based on kernel partial least squares regression (KPLS) [38]. The problem of colorimetric characterization is different from that of color spectral reflectance reconstruction. Although the spectral reflectance of the object surface is a high-resolution expression of the object color, the spectral reflectance of the object surface needs to be combined with the light source to show the color information. And the spectral reflectance of the object surface cannot be directly used for colorimetric characterization and color space conversion; meanwhile, this method uses RMSE for parameter tuning without considering visual color difference, and cannot accurately optimize model parameters for visual color difference. In terms of training sample validity, in 2018, Amiri et al. proposed a weighted nonlinear regression method (WT-NONLIN), which enables commercial digital RGB cameras to be used for spectral and colorimetric color reproduction [39].

The accuracy of color space conversion is constrained by the regression model, the training samples and the feature extraction. The effectiveness of feature extraction is the key to improving the accuracy. We propose a new method for colorimetric characterization of color imaging systems based on KPLS. The method establishes the mapping relationship between the three-channel response kernel function of the color imaging system and the CIE1931 tristimulus values. Through canonical correlation analysis, the direction vector with the strongest correlation between input and output is determined. Based on different direction vectors, a multivariate nonlinear regression model is established. The method considers the relevance between the input vector and the output vector; this method can promote predictive ability when a feature vector is selected. It can effectively solve the multicollinearity problem caused by kernel function expansion, and eliminate irrelevant input variables. It makes the model more concise and stable. It is proved that this method is an effective colorimetric characterization method by using two nested cross-validation.

## 2. Theory and Method

### 2.1. Colorimetric Characterization of Color Imaging System Based on KPLS

**Figure 1:**
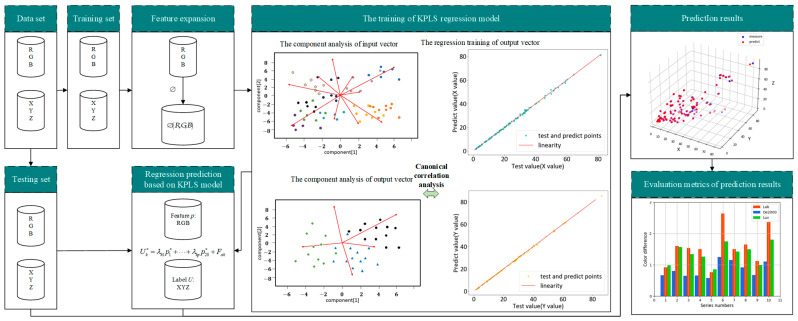
Colorimetric characterization method based on KPLS.

The basic idea of the colorimetric characterization method for a color imaging system is to establish the mapping relationship between the device-dependent space RGB and the device-independent space XYZ, so as to realize the transformation model from RGB to XYZ. In order to obtain the colorimetric characterization mapping from RGB to XYZ, it is usually necessary to use a standard color chart as a measurement sample, and measure its three-channel response values and tristimulus values at the same time, so as to obtain a series of calibration data pairs of XYZ and RGB. In this study, the KPLS method is used for modeling research. Firstly, the RGB dataset is expanded via the kernel function, then the dataset is divided. Next, the PLS regression model is trained. Finally, using color difference as evaluation indicators, the trained model is used to predict on the test set and evaluate the performance of the algorithm. The colorimetric characterization method based on KPLS proposed in this paper is shown in Figure 1.

### 2.2. Kernel Expansion of the RGB Color Value

A series of data pairs of $\left(R,G,B\right)$ and $\left(X,Y,Z\right)$ are obtained by simultaneously measuring the three-channel response values and the three-stimulus values. The input matrix $N={\left(n_{ij}\right)}_{m\times 3}$ and the output matrix $U={\left(u_{ij}\right)}_{m\times 3}$ are composed of m groups of data, where $n_{i}=\left(R_{i},G_{i},B_{i}\right),i=1\cdots m$, $u_{i}=\left(X_{i},Y_{i},Z_{i}\right),i=1\cdots m$. In order to deal with the nonlinear characteristics between variables, we use a nonlinear mapping function ∅ to map the input matrix to the input matrix P in the high-dimensional feature space. That is, $\left(n_{ij}\right)\to \emptyset \left(n_{ij}\right)$. The matrix after dimensionality reduction is shown in Equation (1).

(1) $$P={\left[\emptyset \left(n_{1}\right),\emptyset \left(n_{2}\right),\cdots ,\emptyset \left(n_{m}\right)\right]}^{T}$$

where $\emptyset \left(\right)$ denotes the basis function.

Since the dimension of ∅ can be arbitrarily large or even infinite, we usually define the following kernel matrix K to avoid explicitly using ∅

(2) $$K=PP^{T}$$

where the element $k_{i,j}$ of the $i{\mathrm{th}}^{}$ row and $j{\mathrm{th}}^{}$ column of K is

(3) $$k_{i,j}=\langle \emptyset {\left(n_{i}\right)}^{T},\emptyset \left(n_{j}\right)\rangle =f_{\ker}\left(n_{i},n_{j}\right)$$

where 〈 〉 denotes the inner product operation, and $f_{\ker}$ is the kernel function. In this paper, we use three kernel functions, which are polynomial kernel, root polynomial kernel and Gaussian kernel, to perform dimensionality enhancement; these kernel functions are shown in Equations (4)–(6)

(4) $$f_{\ker-P}\left(n_{i}^{T},n_{j}\right)=\sum_{s=0}^{d}\frac{d!}{s!\left(d-s\right)!}\left({\left(n_{i}^{T},n_{j}\right)}^{s}+s\sum_{t=1}^{s-1}\frac{{\left(n_{i}^{T},n_{j}\right)}^{t}}{t!}+1\right)$$

(5) $$f_{\ker-R}\left(n_{i}^{T},n_{j}\right)=\sum_{s=0}^{d}\frac{d!}{s!\left(d-s\right)!}{\left({\left(n_{i}^{T},n_{j}\right)}^{s}+s\sum_{t=1}^{s-1}\frac{{\left(n_{i}^{T},n_{j}\right)}^{t}}{t!}+1\right)}^{1/s}$$

(6) $$f_{\ker-G}\left(n_{i}^{T},n_{j}\right)=\frac{\exp{\left(-\left\|n_{i}-n_{j}\right\|\right)}^{2}}{2\sigma^{2}},i,j\in m$$

### 2.3. Color Space Conversion Based on KPLS 

To enhance the accuracy of the conversion model between the source color space (RGB) and the target color space (XYZ), we use a kernel function to expand the feature vector for regression. Taking into account the correlation among the expanded feature vectors, we apply the PLS method. PLS regression is related to principal component regression. However, instead of finding a hyperplane of maximum variance between the response and independent variables, it finds a linear regression model by projecting both the predicted and observed variables to a new space. First, the data are normalized.

(7) $$\left\{\begin{array}{l} E_{0}={\left(\left(n_{ij}-\bar{n_{j}}\right)/S_{n_{j}}\right)}_{m\times m}\\ F_{0}={\left(\left(u_{ij}-\bar{u_{j}}\right)/S_{u_{j}}\right)}_{m\times 3} \end{array}\right.$$

P is normalized to $E_{0}$, and U is normalized to $F_{0}$. To consider the correlation between the input and output vectors, we decomposed them and modeled the two direction vectors with the highest correlation [40]. The first component $u_{1}$ is extracted from the independent variable matrix $E_{0}$, and the first component $t_{1}$ of the dependent variable matrix $F_{0}$ is extracted. Namely, $t_{1}=E_{0}w_{1}$, where $w_{1}$ is the direction vector. Because $E_{0}$ is the normalized matrix, $w_{1}$ is the unit vector. It denotes the direction of the first axis in decomposition of $E_{0}$. That is, $w_{1}^{T}w_{1}=1$. Similarly, $u_{1}=F_{0}c_{1}$. The objective function can be described as in Equation (8)

(8) $$\underset{w_{1},c_{1}}{\max}\langle E_{0}w_{1},F_{0}t_{1}\rangle ,\;s.t\left\{\begin{array}{l} w_{1}^{T}w_{1}=1\\ c_{1}^{T}c_{1}=1 \end{array}\right.$$

Using the Lagrange multiplier method to solve, we obtain $w_{1}$ and $c_{1}$. Then we can obtain the components $t_{1}=E_{0}w_{1}$ and $u_{1}=F_{0}c_{1}$. Then, we find the regression equations of $E_{0}$ and $F_{0}$ with respect to $t_{1}$, respectively.

(9) $$E_{0}=t_{1}h_{1}^{T}+E_{1}$$

(10) $$F_{0}=t_{1}r_{1}^{T}+F_{1}$$

where $E_{1}$ and $F_{1}$ are the residual matrices of two regression equations, and the regression coefficient vectors are

(11) $$h_{1}=\frac{E_{0}^{T}t_{1}}{{\left\|t_{1}\right\|}^{2}}$$

(12) $$r_{1}=\frac{F_{0}^{T}t_{1}}{{\left\|t_{1}\right\|}^{2}}$$

To meet the accuracy requirement, we continued to search for the second pair of direction vectors with the highest correlation. The residual matrices $E_{1}$ and $F_{1}$ replace $E_{0}$ and $F_{0}$. We find the second directions of the axis $w_{2}$, $c_{2}$ from $E_{1}$ and $F_{1}$, and then we obtained the second components $t_{2}$, $u_{2}$. Continuing this calculation, if the mathematical rank of P is a, then Equations (13) and (14) can be eventually obtained

(13) $$E_{0}=h_{a}^{T}t_{1}+h_{a}^{T}t_{2}+\cdots h_{a}^{T}t_{a},$$

(14) $$F_{0}=r_{a}^{T}t_{1}+r_{a}^{T}t_{2}+\cdots r_{a}^{T}t_{a}+F,$$

where $h_{1},h_{2}\cdots h_{a}$, $r_{1},r_{2}\cdots r_{a}$ are the regression coefficient.

$t_{1},t_{2}\cdots t_{a}$ can be expressed as a linear combination of the vectors of the standardized vector. Based on Equation (14), we can obtain the regression form as follows

(15) $$F_{0}=r_{1}E_{0}w_{1}^{*}+\cdots +r_{a}E_{0}w_{a}^{*}+F$$

where $w_{a}^{*}=\prod_{j=1}^{h-1}\left(Ι-w_{j}h_{j}\right)w_{a}$, Ι is the unit matrix. Then we can obtain

(16) $$F^{*}=\sum_{j}\lambda_{{}_{j}}^{*}x_{j}^{*}+F$$

where $\lambda_{j}^{*}=\sum_{a}r_{a}w_{aj}^{*}$. Finally, following the standardized inverse process, the regression equation of $F^{*}$ is reduced to the regression equation of P to U. 

**Figure 2:**
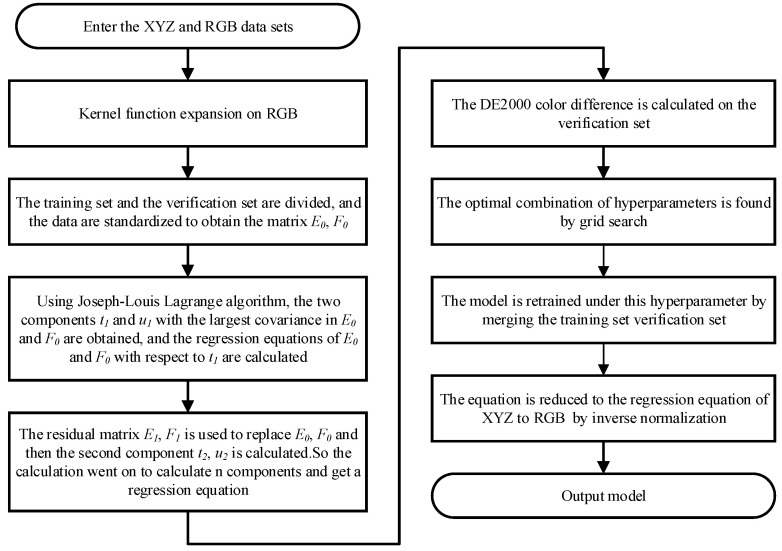
Color space conversion algorithm flow based on KPLS.

The color space conversion algorithm based on KPLS is shown in Figure 2.

### 2.4. Evaluation Metrics

We chose three different color difference formulas according to different application domains to evaluate our model. CIEDE2000 is a widely used color difference formula that considers the needs of different fields [41]. CIELAB color space can better describe the psychological effects of object colors, and it is suitable for materials such as dyes, pigments and inks when the color difference is larger than the visual recognition threshold but smaller than the color difference between adjacent colors in the Munsell system [42,43]. CIELUV is also a relatively perceptually uniform color space, and it is suitable for applications of spectral colors and color vision. The CIELUV color difference formula performs better than other methods when predicting the color difference of illumination stimuli, especially when using a black background [44,45,46]. We used CIELAB, CIELUV and CIEDE2000 color differences to evaluate the generalization ability and prediction accuracy of the KPLS model [47,48,49].

## 3. Experiment

### 3.1. Experimental Scheme

**Figure 3:**
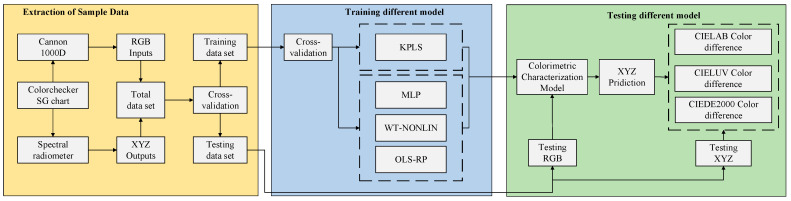
Experimental Scheme.

To validate the proposed model, we first constructed a dataset using the RGB and XYZ values of the color samples. To verify the colorimetric characterization accuracy of the model proposed in this paper, we further divided the dataset into training and testing sets. We trained different colorimetric characterization models based on the training set, and evaluated these models based on the testing set. Finally, we compared the colorimetric characterization effects of different models. The experimental scheme is shown in Figure 3. The color samples used in the experiment are selected from the internationally common ColorChecker SG color chart (X-Rite, Grand Rapids, MN, USA); a D65 light booth (Datacolor, Lawrenceville, GA, USA) was used as the light source. A PR715 spectroradiometer (Photo Research, Syracuse, NY, USA) was used to measure the tristimulus values (CIE XYZ) of 96 non-neutral color patches in the SG color chart. A Canon EOS 1000D was used in manual operation mode. Its resolution was 3888 × 2592 (22.2 mm × 14.8 mm), the ISO was 800 and the F number was 10. When obtaining the color block image, RAW format image was used to capture RGB values. A total of 96 pairs of data were obtained in the experiment, and we divided the 96-group dataset into training and testing sets in accordance with ten-fold cross-validation. 

**Figure 4:**
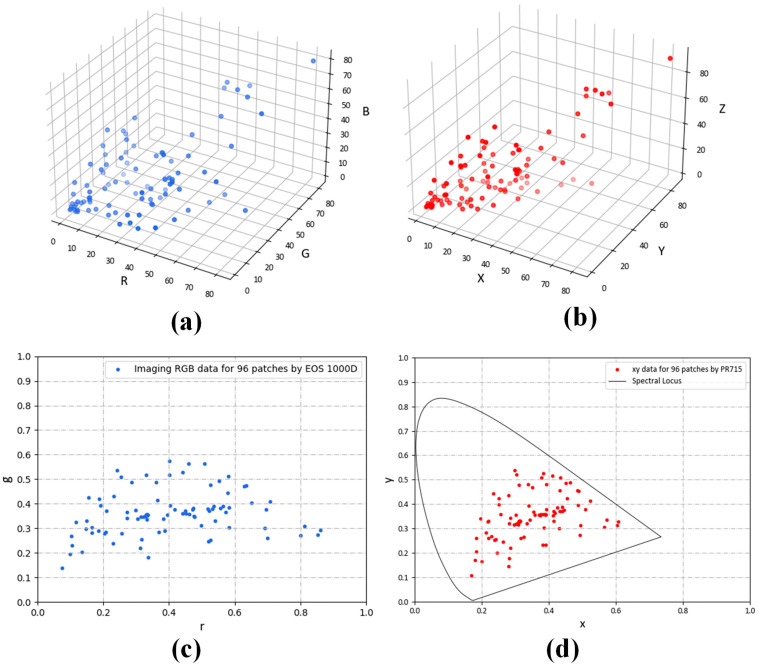
Color and colorimetric distributions of 96 patches collected in our experiment: (**a**) RGB distribution of 96 datasets; (**b**) CIE 1931XYZ tristimulus value distribution; (**c**) device-dependent RGB color space chromaticity distribution; (**d**) CIE1931XYZ chromaticity distribution.

The measured sample dataset is shown in Figure 4. Figure 4a,b show the sample distribution, and Figure 4c,d show the chromaticity distribution.

### 3.2. Correlation Analysis of Input and Output Vectors 

**Figure 5:**
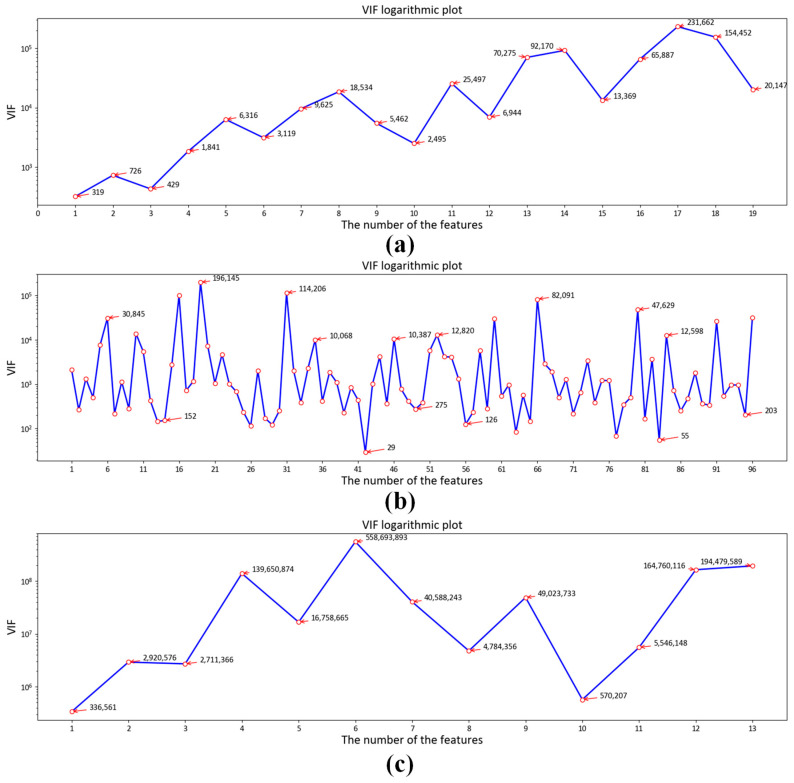
VIF of input feature: (**a**) Based on polynomial kernel function expansion; (**b**) Based on Gaussian kernel function expansion; (**c**) Based on root polynomial kernel function expansion.

The input vector is the kernel function expansion of RGB. Therefore, there is multi-collinearity among input feature variables. The Variance Inflation Factor (VIF) represents the magnification of model parameter estimation variances between non-collinearity and multi-collinearity. It can be calculated with Equation (17)

(17) $$V_{j}={\left(1-R_{j}^{2}\right)}^{-1}$$

where $V_{j}$ represents VIF corresponding to $j{\mathrm{th}}^{}$ variable; $R_{j}^{2}$ is the complex measuring coefficient of regression between dependent variables $x_{j}$ and other independent variables $x_{i}$. The VIF of the input feature variables is shown in Figure 5. If it is more than 100 times larger than that of non-collinearity, the confidence interval is too wide, and the significance test is not credible. Those results reflect the severe collinearity of the R, G and B kernel function expansion. 

**Figure 6:**
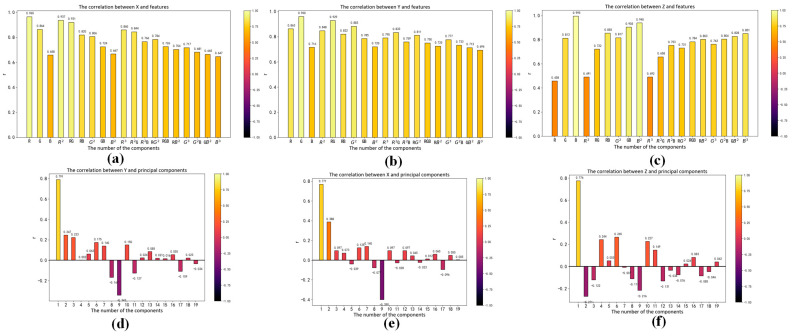
The correlation between XYZ and the polynomial kernel function expansion of RGB: (**a**) Correlation between X and features; (**b**) Correlation between Y and features; (**c**) Correlation between Z and features; (**d**) Correlation between X and features after principal component analysis; (**e**) Correlation between Y and features after principal component analysis; (**f**) Correlation between Z and features after principal component analysis.

**Figure 7:**
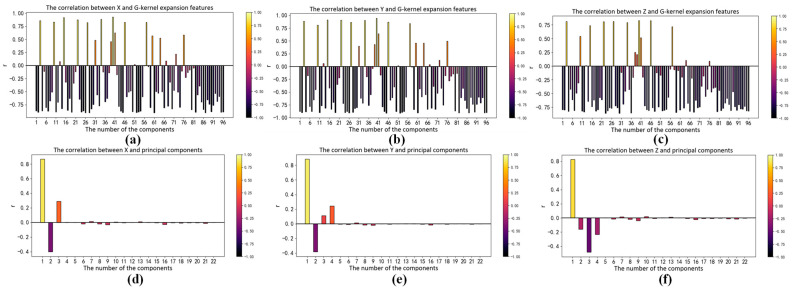
The correlation between XYZ and the Gaussian kernel function expansion of RGB: (**a**) Correlation between X and features; (**b**) Correlation between Y and features; (**c**) Correlation between Z and features; (**d**) Correlation between X and features after principal component analysis; (**e**) Correlation between Y and features after principal component analysis; (**f**) Correlation between Z and features after principal component analysis.

**Figure 8:**
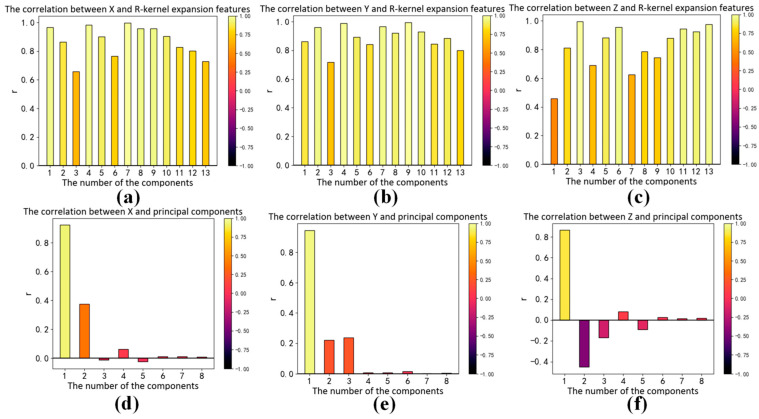
The correlation between XYZ and the root polynomial function expansion of RGB: (**a**) Correlation between X and features; (**b**) Correlation between Y and features; (**c**) Correlation between Z and features; (**d**) Correlation between X and features after principal component analysis; (**e**) Correlation between Y and features after principal component analysis; (**f**) Correlation between Z and features after principal component analysis.

The correlation between input and output vectors directly affects the accuracy of the regression model; therefore, we analyze the RGB kernel function expansion and its correlation with X, Y and Z. The absolute value of the correlation coefficient reflects the degree of correlation. When the absolute values of the correlation coefficient are greater than 0.6, that means high correlation between the two vectors. The Pearson correlation coefficients between each term and X, Y and Z are shown in Figure 6, Figure 7 and Figure 8a–c, respectively. We can see that the input vectors are highly correlated with X, Y and Z, respectively. Moreover, we applied principal component analysis (PCA) to reduce the multicollinearity among terms of input vectors. The VIF of each component after PCA is approximately 1. The Pearson correlation coefficients between each component after PCA and X, Y and Z are shown in Figure 6, Figure 7 and Figure 8d–f, respectively. There is only one component whose absolute value of the correlation coefficient is greater than 0.6. It suggests that most principal components have a low correlation with X, Y and Z. As shown in Figure 5, Figure 6, Figure 7 and Figure 8, RGB kernel function expansion has a high degree of information overlap and low information validity. It represents high multicollinearity, which affects the accuracy of parameter estimation and the robustness of the model. Although PCA can eliminate collinearity, it also reduces the correlation with the dependent variable.

### 3.3. Hyperparameter Selection 

**Figure 9:**
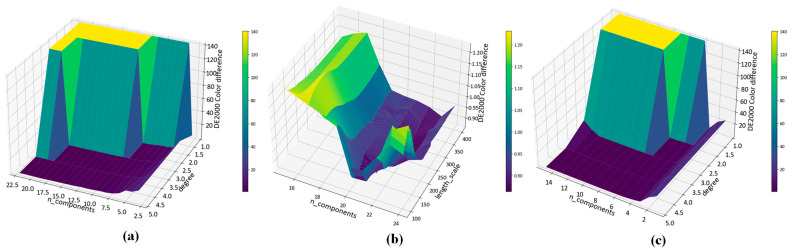
Color difference of CIEDE2000 based on KPLS model under different hyperparameter combinations: (**a**) Based on polynomial kernel function expansion; (**b**) Based on Gaussian kernel function expansion; (**c**) Based on root polynomial kernel function expansion.

**Figure 10:**
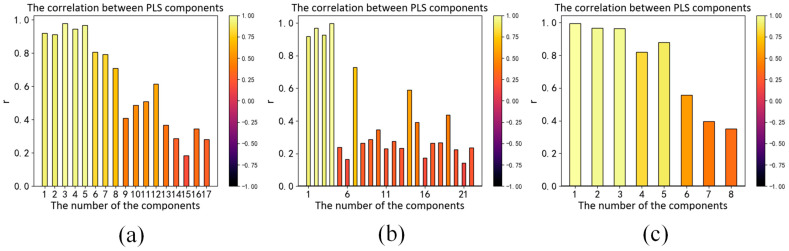
The correlation coefficient between the components of the KPLS model: (**a**) Based on polynomial kernel function expansion; (**b**) Based on Gaussian kernel function expansion; (**c**) Based on root polynomial kernel function expansion.

In this paper, we perform kernel expansion on the input features of the KPLS model using the polynomial kernel function, the Gaussian kernel function and the root polynomial kernel function. We optimize and evaluate their hyperparameters. The hyperparameters include the order of the polynomial, σ which represents the width parameter of the radial basis function and the numbers of components of the PLS model. Unlike the traditional k-fold cross-validation method, we use a nested cross-validation method to select the optimal hyperparameters and evaluate the performance of the model at the same time. We perform two-layer cross-validation. First, we split the training set and test set. Then we split each training set into training set and validation set. The validation set is used for model hyperparameter selection, and the test set is used for accuracy evaluation. The 10-fold cross-validation procedure for model hyperparameter optimization is nested inside the 10-fold cross-validation procedure for accuracy evaluation. The inner loop cross-validation applies grid search to find the optimal hyperparameters within the preset parameter range, so as to minimize the average CIEDE2000 color difference on the validation set; then we use this set of optimal hyperparameters to evaluate the model performance on the test set in the outer loop. Finally, we obtain the CIELAB, CIELUV and CIEDE2000 color differences on the test set. Table 1 shows the optimal hyperparameter combinations selected via the KPLS model based on polynomial kernel expansion, Gaussian kernel and root polynomial kernel expansion in ten training sets. Figure 9 shows the change in CIEDE2000 color difference on the test set under different hyperparameter combinations on the first training set. Figure 10 shows the correlation coefficients between the dependent variable and independent variable obtained via the KPLS model based on polynomial kernel expansion, Gaussian kernel expansion and root polynomial kernel function expansion, respectively. It can be seen that the KPLS model effectively eliminates the problem of multicollinearity between independent variables. 

### 3.4. Experimental Results of This Paper

**Figure 11:**
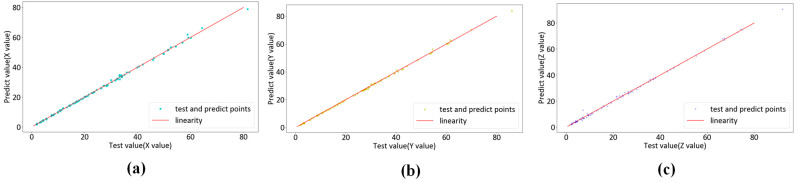
KPLS regression based on polynomial kernel expansion: (**a**) X test data and forecast data pairs; (**b**) Y test data and forecast data pairs; (**c**) Z test data and forecast data pairs.

**Figure 12:**
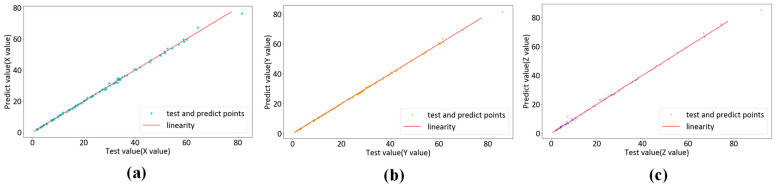
KPLS regression based on Gaussian kernel expansion: (**a**) X test data and forecast data pairs; (**b**) Y test data and forecast data pairs; (**c**) Z test data and forecast data pairs.

**Figure 13:**
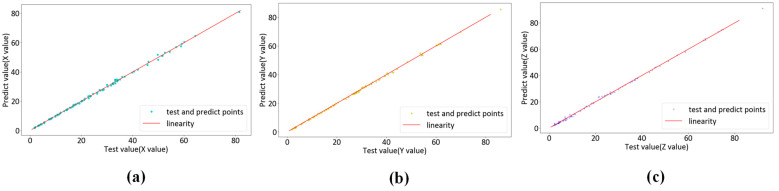
KPLS regression based on root polynomial kernel expansion: (**a**) X test data and forecast data pairs; (**b**) Y test data and forecast data pairs; (**c**) Z test data and forecast data pairs.

To assess the performance of the KPLS model in colorimetric characterization, we use three different color difference formulas—CIEDE2000, CIELAB and CIELUV—to calculate the average color difference on the test set in the outer loop. We collect the test set data from each fold of the cross-validation. We obtain a regression analysis of 96 pairs of actual and predicted data. Figure 11, Figure 12 and Figure 13 show the regression results of the KPLS model based on polynomial kernel expansion, Gaussian kernel expansion and root polynomial kernel expansion on the test set in the 10-fold cross-validation. Table 2 gives the average color difference of the model prediction on the test set of each fold. 

### 3.5. Comparison with Other Methods

**Figure 14:**
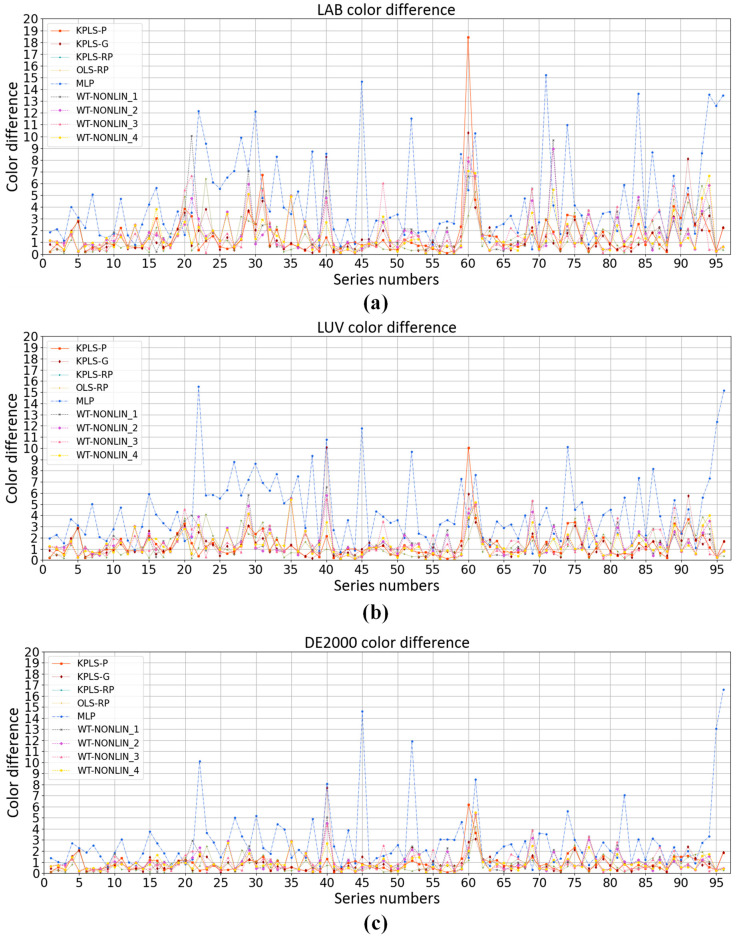
Comparison of colorimetric characteristics of different algorithms in the cross-validation method: (**a**) 96 non-neutral color prediction CIELAB color difference; (**b**) 96 non-neutral color prediction CIELUV color difference; (**c**) 96 non-neutral color prediction CIEDE2000 color difference.

**Figure 15:**
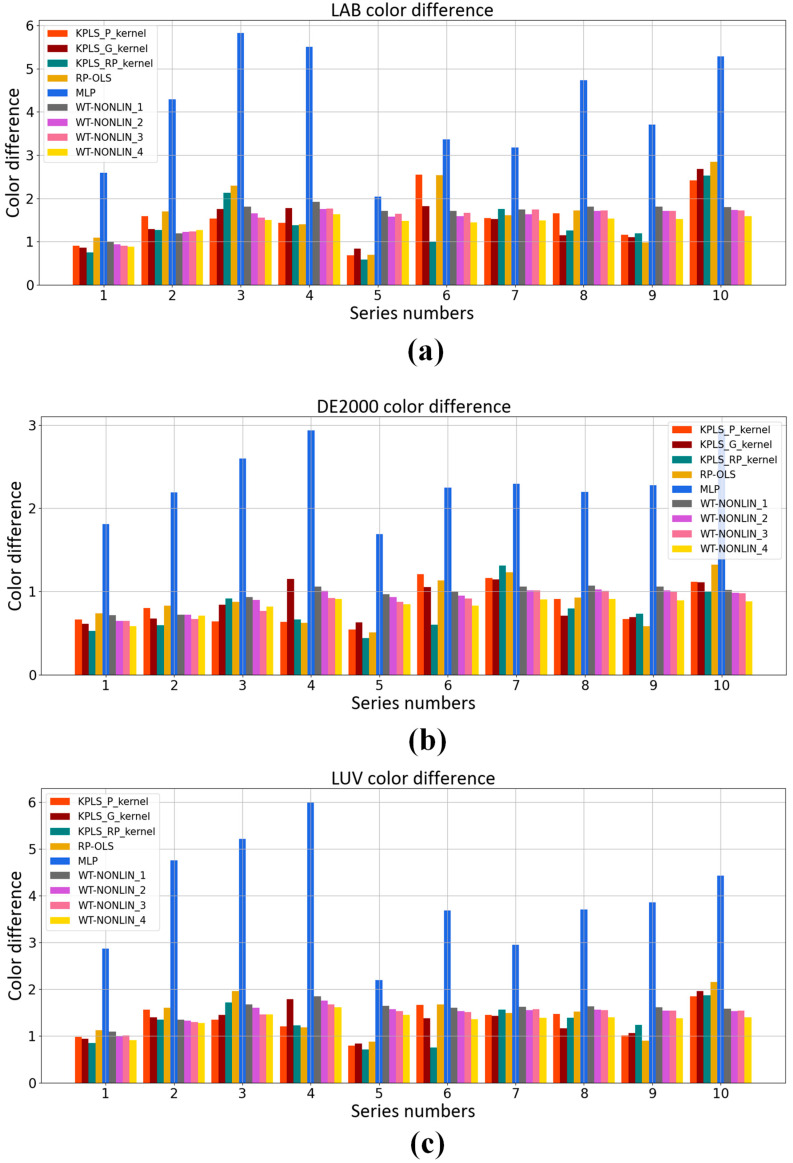
Comparison of average color difference of different algorithms: (**a**) Average CIELAB color difference of ten-fold test data; (**b**) Average CIELUV color difference of ten-fold test data; (**c**) Average CIEDE2000 color difference of ten-fold test data.

Polynomial regression model [21,22] and neural network model [23,24,25,26,27] are used in colorimetric characterization methods commonly. We compare the KPLS model with the weighted nonlinear polynomial regression model, neural network model and root polynomial regression model [32]. The comparison results are shown in Figure 14 and Figure 15. MLP represents the neural network model. In the MLP model, the hidden layer is 10, the learning rate is 0.1 and the learning target is 0.00001. WT-NONLIN_1, WT-NONLIN_2, WT-NONLIN_3 and WT-NONLIN_4 represent the weighted regression model which uses four different formulae for calculating the distance here [39]. RP-OLS represents the root polynomial regression model. We choose 3 as the degree of the polynomial. The same dataset is used in this experiment. After dividing the dataset by nested cross-validation, color differences predicted by each model are compared. In Figure 14, the ten test sets are collected together. There are 96 samples. We compare the average color differences of different models under the same sample. In Figure 15, we compare the average color differences of different models of each fold test set. The CIELAB, CIELUV and CIEDE2000 color differences of the test set are calculated and compared. The experimental results are shown in Table 3. Table 3 shows the average color difference of the model prediction on the test set of each fold with different algorithms.

## 4. Discussion

**Figure 16:**
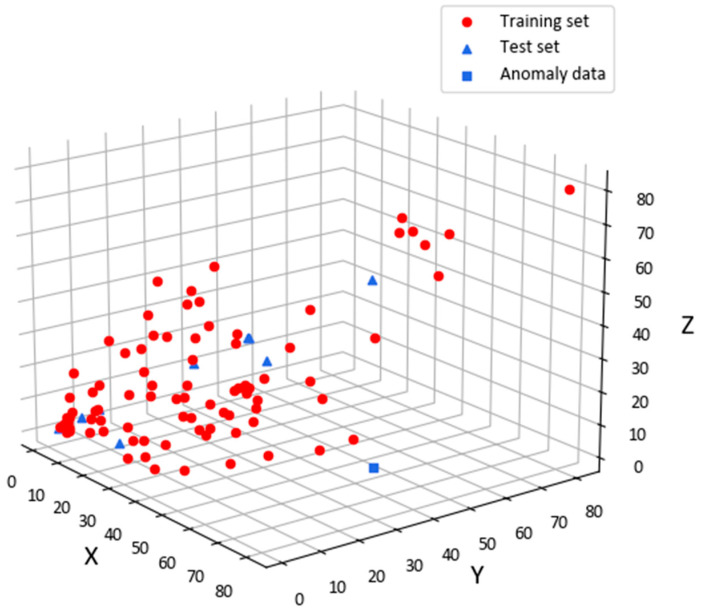
Test set distribution where the outliers are located.

We observed a large color difference point in the colorimetric characterization results of the KPLS model and the weighted nonlinear polynomial regression model. This point is the 60th pair of data, corresponding to the 6th fold of the ten-fold data. The sample distribution of the training and test sets for this group of data are shown in Figure 16. The red points are the training set; the blue points are the test set, and the blue square point is the 60th pair of data. We can see that these data are far away from the training set samples in the test set. They belong to the extrapolation prediction of the model. They exhibit a large difference in comparison with the data in the training set, so we cannot accurately predict them. Therefore, how to increase the coverage range of the color gamut of the training dataset or improve the accuracy of the model to enhance the prediction ability of the extrapolation points is the next research direction.

## 5. Conclusions

This paper studies a colorimetric characterization method for color imaging systems based on KPLS. The method uses device-dependent RGB space as input features and expands the feature vectors with kernel functions. According to feature analysis and canonical correlation analysis, a KPLS colorimetric characterization model for color imaging systems is established. The hyperparameters of the model are determined via nested cross-validation and grid search, and the color space conversion model is constructed. The experimental verification is carried out by using a Canon 1000D commercial digital camera to shoot the SG color card, and CIELAB, CIELUV, CIEDE2000 color differences are used for evaluation. The results show that the color difference of the KPLS colorimetric characterization model based on root polynomial function expansion is better than that of the weighted nonlinear regression model, the neural network model and the root polynomial regression model. The colorimetric characterization method for color imaging systems based on KPLS is an effective method with good prediction accuracy and nonlinear fitting ability, which can support the cross-media color management of color imaging systems well.

## Figures and Tables

**Table 1:** The Hyperparameter optimization results of KPLS model in cross-validation.

The Series of Fold	P-Kernel			G-Kernel			RP-Kernel		
	Order	Components	DE2000	σ	Components	DE2000	Order	Components	DE2000
1	3	17	0.862	176	21	0.863	3	8	0.752
2	3	18	0.895	285	22	0.858	3	8	0.756
3	3	17	0.897	196	21	0.880	3	8	0.742
4	3	18	0.947	400	20	0.940	5	11	0.759
5	3	17	0.941	267	22	0.896	5	12	0.764
6	3	17	0.794	236	23	0.882	5	8	0.769
7	4	18	0.825	230	22	0.773	3	8	0.670
8	3	18	0.838	226	22	0.749	3	9	0.699
9	3	18	0.898	225	22	0.861	5	11	0.720
10	4	17	0.840	245	23	0.738	5	8	0.707

**Table 2:** Model average color difference per fold test set.

The Series of Fold	P-Kernel			G-Kernel			RP-Kernel		
	LAB	LUV	DE2000	LAB	LUV	DE2000	LAB	LUV	DE2000
1	0.907	0.975	0.666	0.855	0.933	0.613	0.746	0.848	0.527
2	1.583	1.558	0.801	1.294	1.401	0.676	1.273	1.344	0.597
3	1.535	1.351	0.640	1.755	1.444	0.844	2.130	1.718	0.918
4	1.429	1.207	0.635	1.772	1.784	1.150	1.376	1.221	0.665
5	0.681	0.798	0.541	0.834	0.835	0.631	0.581	0.714	0.442
6	2.550	1.666	1.206	1.823	1.372	1.053	1.007	0.753	0.603
7	1.542	1.452	1.188	1.517	1.429	1.144	1.749	1.557	1.311
8	1.655	1.471	0.898	1.146	1.164	0.711	1.258	1.391	0.799
9	1.154	1.010	0.665	1.099	1.056	0.696	1.194	1.232	0.733
10	2.411	1.847	1.116	2.684	1.954	1.113	2.522	1.865	1.003

**Table 3:** The average color difference of different algorithms.

Model		CIELAB Color Difference	CIELUV Color Difference	CIEDE2000 Color Difference
KPLS	P-kernel	1.5447	1.3335	0.8356
KPLS	G-kernel	1.4779	1.3372	0.8631
KPLS	RP-kernel	1.3836	1.2643	0.7598
RP-OLS [32]		1.4221	1.2933	0.7775
MLP		4.052	4.4166	2.8895
WT-NONLIN formula 1 [39]		1.7977	1.5839	1.0207
WT-NONLIN formula 2 [39]		1.7272	1.5343	0.9858
WT-NONLIN formula 3 [39]		1.7242	1.5429	0.9799
WT-NONLIN formula 4 [39]		1.5878	1.4017	0.8847

## Data Availability

The data that support the findings of this study are available on request from the corresponding author.
